# Surgical resection of a renal cell carcinoma involving the inferior vena cava: the role of the cardiothoracic surgeon

**DOI:** 10.1186/1749-8090-5-103

**Published:** 2010-11-05

**Authors:** Haralabos Parissis, Mohammad Taukeer Akbar, Michael Tolan, Vincent Young

**Affiliations:** 1 Royal Victoria Hospital, Grosvernor Rd, Belfast, BT12 6BA, Northern Ireland; 2 Essex Cardiothoracic Center, Basildon & Thurrock University Hospital, Essex, UK; 3 Cardiothoracic Department, St James Hospital, Dublin, Ireland

## Abstract

**Background:**

The techniques for the resection of renal tumors with IVC extension are based on the experience of individual units. We attempt to provide a logical approach of the surgical strategies in a stepwise fashion.

**Methods:**

Over 6-years 9 patients with renal cell carcinoma invading the IVC, underwent surgery. There were 6 males. The extension was at level IV in 4 and III in 5 cases. CPB used in 8 and hypothermia and circulatory arrest in all patients with level IV disease. The results and an algorithm of the plan of action, as per level of extension are presented.

**Results:**

Plan of action: For level I-II disease: No Cardiothoracic involvement, For level III: Cardiopulmonary Bypass (CPB) & control of the cavo-atrial junction. For level IV: use of brief periods of Circulatory Arrest & repair of the Cavotomy with a pericardial patch. Postoperative morbidity: prolonged ICU stay, 3 patients (33.3%); tracheostomy, 1 (11.1%); Sepsis, 2 (22.2%); CVA 1, (11.1%). Mortality: 2 patients (22.2%)

**Conclusions:**

Total clearance of the IVC from an adherent tumor is important, therefore extensive level IV disease presents a surgical challenge.

We recommend CPB for level III and brief periods of Total Circulatory Arrest (TCA) for level IV disease.

## Background

Inferior Vena Cava (IVC) involvement in patients undergoing surgery for renal cell carcinoma (RCC) is rare (4-8%) [1]. The overall 5 year survival following successful resection can be up to 40 - 50% [2,3], therefore one should not preclude surgical therapy in this group of patients [4].

The level of the IVC involvement as defined in the literature [1,3,4], dictates the surgical strategies and mandates the development of a plan of action that should be safe, reproducible and reliable.

Favorable outcome in patients with non-metastatic renal carcinoma and IVC involvement correlates with complete clearance of the IVC from tumor-thrombus. This principle sometimes can only be achieved following an optimal exposure of the infra & supra hepatic IVC concomitantly with clearance of the IVC -right atrial junction. Furthermore prevention of tumor disruption and pulmonary embolism has to be considered during thrombectomy & manipulation of the diseased cava.

The guidelines regarding the various techniques for the resection of RCC with IVC extension are very scattered in the literature. In this article we attempt to provide a systematic approach of the cardiothoracic surgical strategies in a stepwise fashion.

## Methods

**Figure 1:**
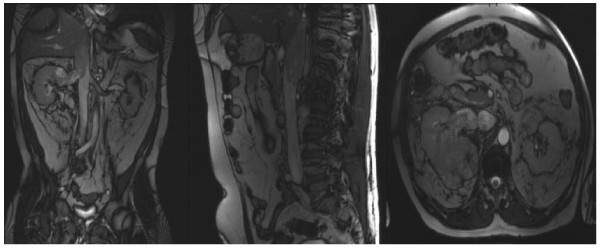
**MRI images of a level IV disease**.

**Figure 2:**
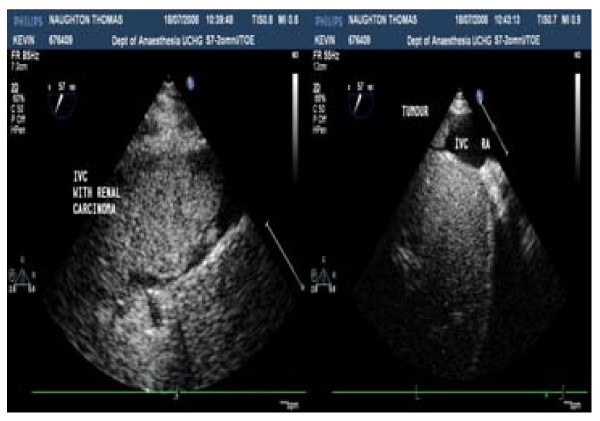
**Echo images of tumor extending into the IVC**.

Over 6-years 9 patients with RCC invading the IVC, underwent surgery. There were 6 males. The extension was at level IV in four(4) and III in five(5) cases. Cardio Pulmonary Bypass was used in eight(8) patients and hypothermia and circulatory arrest in all patients with level IV disease. Abdominal MRI (Figure 1) is useful to determine the extent of IVC involvement with tumor/thrombus. Peri-operative Trans-Oesophageal Echo (Figure 2) provides information's regarding the amount of adherence, supra-hepatic extension and mobility of the tumour. Multidisciplinary approach is needed. Metastatic disease is a contraindication for surgical therapy and has to be ruled out. The patients characteristics are present in appendix 1.

### Surgical Approach

Mobilisation of the affected kidney with retroperitoneal lymphadenectomy is performed first. For level I-II disease cardiothoracic involvement is not necessary. Limited cavotomy with the brief use of an intermittent Caval clamp above and below the lesion is usually adequate. The need for cardiac surgical involvement is usually contemplated when the tumor/thrombus is extending up to level III. We favour a standard midline laparotomy and assessment of resectability of the renal tumour.

Following sternotomy, institution of CPB is achieved using a split venous cannula: Superior Vena Cava & Right femoral vein. Control of the cavo-atrial junction is considered in order to avoid tumour embolization. Bulky disease extending into the right atrium may be better controlled by splitting the diaphragm through the central tendon towards the IVC. This manoeuvre, enables extension of the Right atrial incision towards the IVC for direct resection of severely adhere tumours (ie. Patient number 3).

The porta hepatis is dissected so that the liver blood supply could be briefly interrupted (Pringle manoeuvre: occlusion of blood inflow to the liver) during cavotomy to further facilitate bloodless surgical field. Furthermore, by applying a cross clamp on the sub-diaphragmatic aorta during caval extirpation of the tumour, bloodless operative conditions could be achieved.

Level IV involvement presents a challenge; the disease extends into the RA with various degrees of infiltration and adherence into the wall of IVC. Under those circumstances the use of Total Circulatory Arrest (TCA) has become the centre of an argument. The patho-physiological sequelae of the use of TCA are balanced against the risk of a suboptimal tumour clearance. We, like others believe that with such extension of the disease the wall of the IVC is infiltrated by tumour and unless a complete bloodless field is instituted, only by blunt dissections, it is impossible to achieve complete clearance.

**Figure 3:**
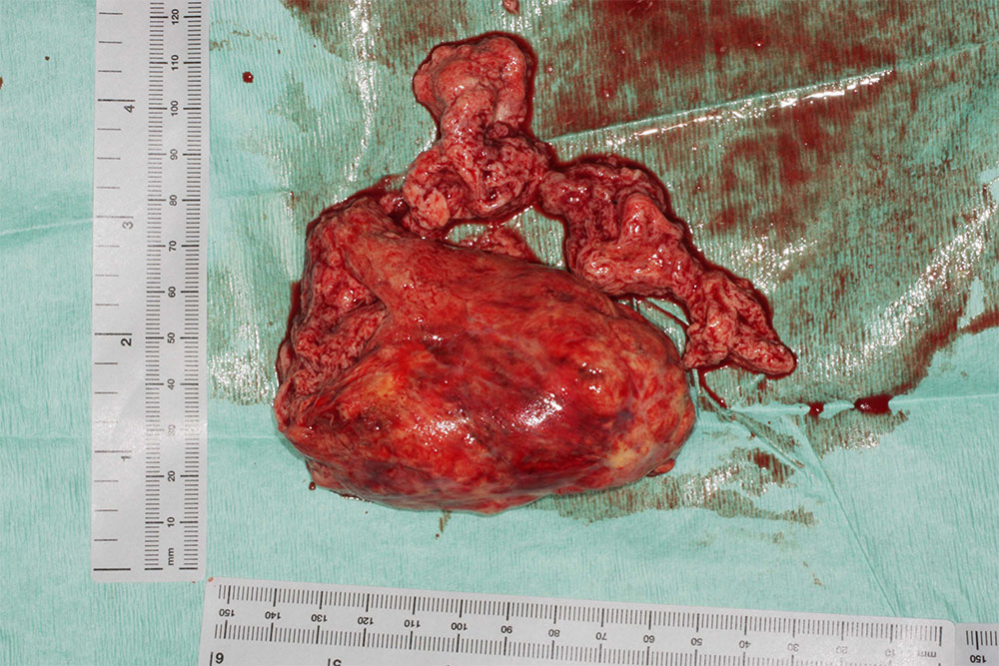
**Renal cell carcinoma invading the upper pole of the kidney with tumor propagating into the IVC**.

**Figure 4:**
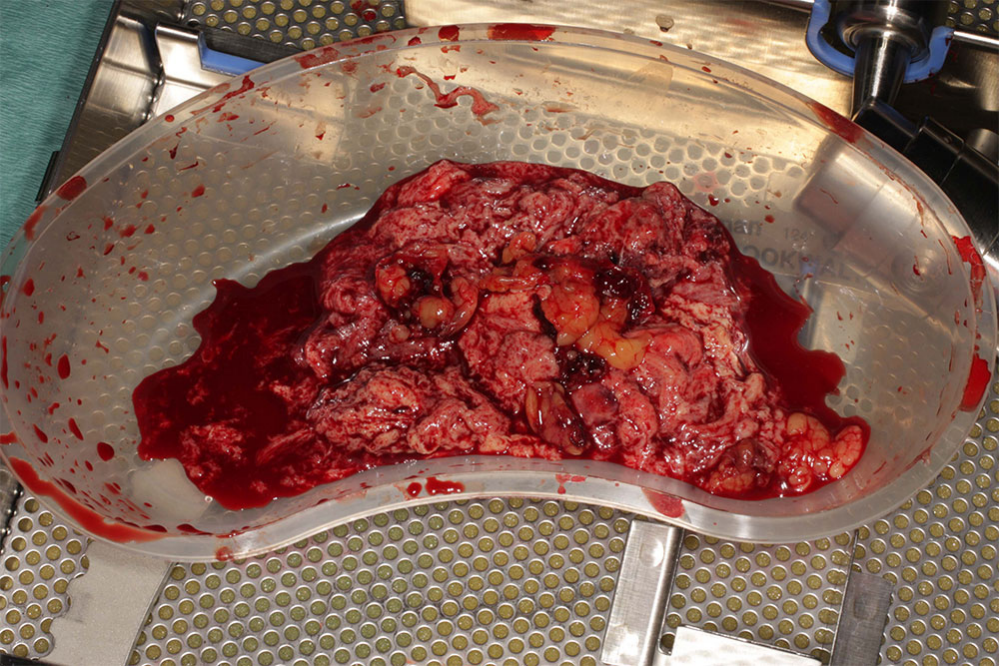
**Direct removal of the tumor mass**.

**Figure 5:**
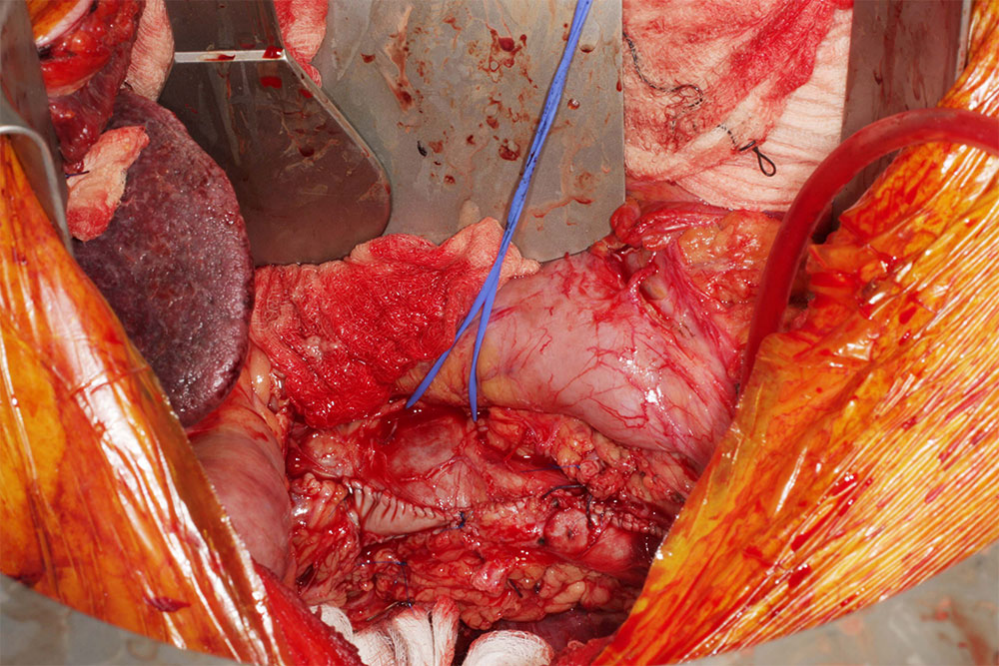
**Closure of the IVC with a pericardial patch**.

Therefore for level IV extension of the tumour or for suspected "suboptimal thrombectomy" for level III disease we advocate brief period of TCA. During the cooling period in an arrested heart the RA is opened and tumour mobilization around the ostium of the IVC is carried out. Endarterectomy knifes further facilitate optimal extirpation of the tumour by negotiating anatomical planes of excision. During TCA the cava is incised up to 10 cm cephalad in a longitudinal fashion taking care to include with the specimen the origin of the renal vein which is usually involved with the tumour. Clearance of the luminal deposits of the IVC using sharp and blunt dissections could be then carried out under direct vision. Having mobilised the tumour proximally at the IVC- RA junction, final extraction is usually achieved in continuity with the nephrectomy specimen (Figure 3). Furthermore, tumour embolization to the lungs is avoided. This process provides a controlled bloodless environment for facilitation of complete tumour clearance (Figure 4). Always the cavotomy is repaired with the use of a pericardial patch (Figure 5), in order to avoid narrowing of the cava. An algorithm of the plan of action, as per level of extension is depicted in appendix 2.

## Results

### Outcome

During the beginning of this program, Venovenous bypass was used in one patient (number 7) with level III disease. However the technique was deem cumbersome and unsatisfactory, mainly due to excessive blood in the surgical field, resulting in suboptimal exposure.

Cardio Pulmonary Bypass was used in eight(8) patients and hypothermia and circulatory arrest in all patients with level IV disease.

The operative time range from 3 hours 52 minutes to 9 hours 36 minutes. Estimated blood loss was 1850 mL (range 950 to 3800 mL). Blood and blood product requirement was high (7 out of nine patients). The average blood transfusion was 2 units of red Blood Cells (range between 1 and 4 Units). Blood products were used in all four patients following hypothermia and circulatory arrest. Cell-saving techniques used routinely in our institution.

Transient inotropic support by means of Dopamine and Noradrenaline was used in 5 patients. Average intensive care unit length of stay was 19 days (range, 1 to 164 days). In three (3) patients (33.3%) the ICU stay was prolonged. Furthermore one (1) patient required a tracheostomy (11.1%). Two patients developed septicemia (one MRSA positive) and one patient develop a CVA. Two patients died; one from septicaemia post-operative day 55 and one from multiple organ failure post operative day 164. The mean size of the renal mass was 5.2 cm (range, 3.5 to 11.2 cm). Histological examination showed renal cell carcinoma of clear type in 8 patients and papillary type in 1 patient. Lymph node metastasis was detected in 2 patients.

Two of the discharged patients were lost to follow up. Of the remaining five patients, 2 have had tumor recurrence and one had pulmonary metastasis at 2 years, on follow up chest X Ray. Those 3 patients were referred for adjuvant chemotherapy. The cumulative postoperative follow-up of the remaining two patients was 45+/-11 months. They were alive at the last follow up and free of recurrence.

## Discussion

Metastasis has occurred in 34.6% of the patients with RCC and luminar propagation of the tumor into the IVC [5]. Furthermore, as per the same authors, micrometastasis is taken place in 11.1% of those patients. Therefore, only half of the patients with level III-IV disease would be free of distal spread and subsequently would benefit from an operation. Palliative resection to control polycythemia and paraneoplastic syndromes in patients with metastatic disease, is questionable.

Level I and II is probably the commonest entity occurring in 60-65% of the cases and usually treated by local resection. According to Lubahn et al [6] approximately 50% of the patients with renal tumors involving the IVC, warrant cardiothoracic involvement. Furthermore the overall incidence of extensive IVC disease involving the right atrium according to Bissada et al [5] & Hermanek et al [7] is around 27.7%.

It has been postulated that the involvement of the IVC in RCC is generally not a vascular invasion by the malignancy [8]; one could argue however, that following removal of the thrombus-tumor from the IVC, invariably, an area is found that indicates sub-endothelial invasion. In addition, in 12.9% of the patients in Bissada et al series [5] the IVC wall was invaded by tumor.

Suprahepatic extension of the tumor (level III disease) poses a challenge, especially when the tumor is densely adhering to the Venus wall or when the hepatic veins contain propagating segments of tumor. Budd-Chiari syndrome, is an extreme form of hepatic venous stasis resulting from occlusion of the major hepatic veins or the supra- hepatic IVC from various malignant causes, with renal cell carcinoma being the most common. A hepatic vein obstruction that causes Budd-Chiari syndrome, is an adverse feature. Under such conditions, bleeding diathesis is accelerated; this is due to Liver congestion with reduce "synthetic function" and also portal hypertension with the development of porta-caval collaterals.

Generally for level III disease some institutions [9] favor cavotomy without the use of CPB [10] or with the use of venous-venous bypass [11,6]. The latter group in a large series of patients concluded that the need for invasive cardiovascular procedures increased the risk of perioperative complications. The advantages of using veno-venous bypass are restoration of hemodynamic instability during venal clamping and the fact that there is no need for systemic heparinization. However one would argue that without CPB and possibly without additional maneuvers to reduce the venus return (such as Pringle maneuver, clamping of the abdominal aorta, the superior mesenteric artery or the contralateral renal artery) bloodless field cannot be achieved during cavotomy; furthermore the imposed hemodynamic instability at the time, has another adverse impact: the surgeon is "pushed" to complete the extirpation of the thrombus against the time. That can rather lead to de-bulking of the tumor. It could also lead to dislodgment of tumor material and subsequent pulmonary embolism. Therefore, for level III disease, besides CPB we would also favor the approach reported by Chowdhury et al [12] whereby intermittent cross clamp of the sub-diaphragmatic aorta is applied. This brief maneuver would further optimize the conditions for a bloodless surgical field.

In the situation where the IVC is fully occluded by the tumor in level III disease, then probably the patient may tolerate clamping of the IVC at the junction with the RA (under TOE guidance) without significant hemodynamic compromise. Under those circumstances, one could debate that CPB is not necessary. Nevertheless, one should bear in mind the theoretical risk, that de-balking of the tumor increases the incidence of local recurrence.

Five patients in our series had level III disease (Three patients had Right side RCC). Venovenous bypass was used in one patient. The tumor was removed satisfactory, however hemodynamic instability and access was deemed cumbersome. Complications with Venovenous bypass [6] and difficulty in accessing the hepatic veins and suprahepatic cava lead us to abandoning this procedure.

For level IV disease with tumor extension in the right atrium controversy still exists as regarding the need for Total Circulatory Arrest (TCA). Sosa et al [13] has reported a poor survival for patients with level IV disease. Cerwinka et al [14] advocates excision of supra-diaphragmatic tumors off pump with no TCA. In contrary, Chiappini et al [15] and Mazzola et al [16], claim that the use of TCA provides a safe technique for removing the tumor thrombus in a bloodless field, and has good early and long-term results. We, like others [17] believe that when the tumor thrombus is invading the caval wall or reaches the right atrium-ventricle then TCA becomes a necessity. We reckon that this approach has improved the safety and efficacy of a difficult surgical undertaking by facilitating controlled dissection, providing a bloodless field, and reducing the risk of tumor embolization. The high postoperative morbidity reported by various groups [13,15] is reflecting the preoperative compromise health status of this group of patients and possibly the use of circulatory arrest. According to Cooper et al [18] the use of TCA increases up to 40% the risk of complications and also adds up, on the peri-operative mortality. Furthermore as per Schimmer et al [17] the risk of bleeding (at least theoretically) could be exponentially higher due to: 1) profound hypothermia itself 2) extended bypass time as a result of cooling-rewarming, and 3)the fact that those patients have undergone extensive retroperitoneal dissections and have accessory high pressure venous collaterals due to the IVC obstruction.

For all those reasons aforementioned, a single institutional approach [19] advocates in selected cases of renal cell carcinoma with level IV IVC extension, resection of the tumor without sternotomy, CBP, or DHCA. This technique however has limitations ([19] Invited commentary).

The need for extensive surgery with relative good outcome has been outlined from various groups. According to Tanaka et al [2] and Yazici and associates [20] the length of tumor extension is not an incremental risk factor for adverse survival. Likewise Chiappini et al, [15] states that the tumor extension into the IVC to whatever degree is not associated with an adverse prognosis, provided a complete resection is advocated [21].

Complete resection of the entire tumor is mandatory for a reasonable attempt at a long survival, as demonstrated by Nesbitt and colleagues [9] and Hatcher and colleagues [22], where no patients with incomplete local resection survived to 5 years. Following the same principle we favor "Controlled Cavotomy" whereby the interior of the IVC can be adequately inspected in a bloodless surgical environment.

Finally, survival is also associated with the tumor characteristics (grade of tumor cells) and lymph node involvement [2]. Throughout the literature the overall 5 year survival is been reported to be between 40 to 50% overall [3,23,18,24].

Five patients in our series were followed up. There was lymph node involvement at the initial specimen of the two patients, that had local recurrences at 2 years. Of the remaining 3 patients, one had pulmonary metastasis at 2 years, and 2 patients were alive at 4 years and free of recurrence.

## Conclusions

In summary, RCC with advance IVC involvement poses a surgical challenge. During this report we eluded on the pros and cons of the various approaches. In keeping with the principles for local clearance one should consider: multidisciplinary approach with proper pre-operative evaluation of the extension of the tumor, optimal control of hemodynamic conditions during cavotomy, ability to visually assess the extent of the tumor invasion, avoidance of tumor fragmentation and embolization and repair of the IVC without narrowing of the vessel.

Finally in this paper, although the number of patients reported is small, we have attempted to provide a clear strategy for tackling a difficult and unusual entity.

## Consent

Written informed consent was obtained from the patients for publication of the series and accompanying images. A copy of the written consent is available for the review by the Editor-in-Chief of this journal.

## Competing interests

The authors declare that they have no competing interests.

## Authors' contributions

HP conceived of the study and wrote the manuscript with the help of MTA. MT made valid corrections, VY organized and overlooked the progress of the manuscript and advised on valuable points. All authors read and approved the final manuscript.

## Appendix 1: Patients' characteristics

**Table 1:** Patients' characteristics

Sex	Pre-Op Creatinine	Hgt (cm)	Weight (kg)	Euroscore	Operation-Findings	CPB (min)	Cross Clamp Time (min)
m	175	182	85	4	left kidney tumor Level IV	111	43

m	132	182	90	7	Lt Kidney tumor Level III	51	17

f	108	154	60	7	right renal tumor Level IV	101	37

m	124	178	76	5	right renal tumor, Level III	22	0

f	79	166	76	3	right renal tumor, Level III	36	0

m	144	183	80	4	Right kidney tumor Level IV	89	19

m	104	170	106	2	right renal tumor, Level III	0	0

f	103	155	72.5	5	left kidney tumor Level IV	75	25

m	86	180	66	2	left renal tumor, Level III	13	0

## Appendix 2: Surgical steps as per level of IVC involvement by tumor

**Table 2:** Surgical steps as per level of IVC involvement by tumor.

**Surgical steps - IVC involvement**
**↓**
**Level I-II (60% of the cases) No cardiothoracic involvement/Cardiothoracic "back up" only**
**↓**
Level III & IV disease mandates Cardiothoracic involvement
**↓ ↓**
**LEVEL III (12-15% of the cases)**
**LEVEL IV (25% of the cases)**
**CPB, Pringle manoeuvre and if necessary**
**Always use of CPB and brief period of cross clamp of sub-diaphragmatic aorta TCA**
**If suboptimal thrombectomy, then brief TCA**
